# Phosphatase and Tensin Homolog Mutation in Immune Cell Infiltration and Clinicopathological Features of Low-Grade Gliomas

**DOI:** 10.3389/fmolb.2020.562416

**Published:** 2020-12-10

**Authors:** Peng Feng, Zhenqing Li, Yuchen Li, Yuelin Zhang

**Affiliations:** ^1^Xi’an Medical University, Xi’an, China; ^2^Research Center of Clinical Medicine, Affiliated Hospital of Nantong University, Nantong, China; ^3^Hengyang Medical College, University of South China, Hengyang, China

**Keywords:** phosphatase and tensin homolog, prognosis, mutation, low-grade gliomas, gene

## Abstract

The mutation of phosphatase and tensin homolog (*PTEN*) genes frequently occur in low-grade gliomas (LGGs) and are deeply associated with a poor prognosis and survival rate. In order to identify the crucial signaling pathways and genes associated with the *PTEN* mutation, we performed bioinformatics analysis on the RNA sequencing results, which were obtained from The Cancer Genome Atlas database. A total of 352 genes were identified as differentially expressed genes (DEGs). The gene ontology (GO) and Kyoto Encyclopedia of Genes and Genomes (KEGG) analysis suggested that the DEGs were significantly enriched in categories associated with cell division and multiple metabolic progressions. The histological stage was significantly associated with *PTEN* expression levels. In addition, the *PTEN* mutation was associated with an abundance of *B* cells, neutrophils, macrophages, dendritic cells, and *CD8+ T* cells during tumor infiltration. The results showed that patients with LGGs harboring the *PTEN* mutation had a poor prognosis and more serious immune cell infiltration occurred depending on the mRNA expression level. These results demonstrated that multiple genes and signaling pathways play a key role in LGG from low grade to high grade, and are associated with *PTEN* mutations. In this study, we outlined an approach to assess the influence of *PTEN* mutations on prognosis, overall survival, and messenger RNA (mRNA) expression. Our results provided alternative strategies for the personalized treatment of patients with LGGs harboring the *PTEN* mutation.

## Introduction

Gliomas, as a kind of common craniocerebral tumor, can be divided into four grades based on the 2007 World Health Organization classification of tumors. Grade I and II are low-grade gliomas (LGGs), while Grade III and IV are high-grade gliomas (Louis et al., 2007). A clinical investigation into malignant LGGs found that the overall survival of LGGs is significantly higher (2–4 years) than high-grade gliomas (∼15 months), which are highly aggressive tumors and exhibit significant aggression (Buckner et al., 2016). Reported by a variety of literature, too many genes anticipate the signal pathway of a drug resistance response (Andersson et al., 2004; Calatozzolo et al., 2012). To precisely treat LGGs patients, it is of importance to provide personalized genetic information and expression correlations, because personnel treatments can provide precise therapeutic strategies based on specific genetic conditions. Consequently, the identification of the underlying biomarkers of disease progression and the underlying target gene are the prerequisite for personalized treatments.

The phosphatase and tensin homolog (*PTEN*) gene is a multifunctional tumor suppressor, which contains a catalytic domain and a tensin-like domain (Li et al., 1997; Helseth et al., 2010). Owing to the tumor suppressing functions of the *PTEN* gene, it has been found to mutate with high frequency in several types of carcinomas, including LGGs (Helseth et al., 2010; Johnson and O’Neill, 2012). To promote the cell proliferation of cancer, the key node target is Akt, in which the PTEN protein inhibits PI3K/Akt signaling and then activates the P21 protein (Mu et al., 2020). Although the variation in *PTEN* expression levels may correlate with the LGG tumorigenesis (Wiencke et al., 2007), these expression levels have clinical significance and can be used as prognostic biomarkers. Some studies have reported that *PTEN* is activated through AKT-independent (protein kinase B) mechanisms (McGirt et al., 2005). Patients harboring the *PTEN* mutation exhibit increasing alterations of multiple signaling pathways and cellular metabolism compared with those harboring the wild-type *PTEN* gene (Steck et al., 1997). Thus, a variation in the *PTEN* status may affect the tumor progression by regulating the immune microenvironment (Best et al., 2018; Wu et al., 2018). And, the disease prognosis and immune cell infiltration are highly associated with the immune microenvironment (Ino et al., 2013), as well as resistance or sensitivity to treatment measures (Norton et al., 2019). However, the importance of *PTEN* status in LGG progression and the molecular mechanism is still unclear.

In this study, we analyzed the RNA sequencing data of LGG patients, obtained from The Cancer Genome Atlas (TCGA) database. By performing the identification of differentially expressed genes, the molecular functions and correlation with LGG progression were analyzed using GSEA analysis. After the enrichment of differentially expressed genes, the association between differentially expressed genes and immune cell infiltration was further analyzed using the TIMER database, which elucidated the effect of the *PTEN* mutation on the tumor-related genes and signaling pathways.

## Materials and Methods

### Gene Set Enrichment Analysis

The RNA-seq database of LGG patients was obtained from The Cancer Genome Atlas (TCGA) database^1^, which included 516 cases. After the classification of differentially expressed genes, gene set enrichment analysis (GSEA) was used to identify the biological functions of the differentially expressed genes (DEGs) based on their biological status. Furthermore, the enriched signal pathways of LGG patients with or without the *PTEN* mutation were obtained. Enrichment results with a cut-off value of false discovery rate (FDR) < 0.25 and a *p*-value < 0.05 were identified to be as significant. The hazard ratio of LGG patients, including age, gender, PTEN status, and grade, were performed using the Cox proportional-hazards model of the R software.

### Identification of Differentially Expressed Genes

In this study, the R software (version 3.5.2) containing the bioconductor software package (EdgeR) was used to identify the differential gene expression in LGG patients harboring various *PTEN* mutations compared with wild-type patients (Robinson et al., 2010; McCarthy et al., 2012). The identification criteria for the DEGs were as follows: *P*-value and FDR < 0.05; |log2FoldChange| ≥ 1.0.

### Pathway Enrichment Analysis of Differentially Expressed Genes

GSEA analysis was performed to ascertain the effect of differentially expressed genes on signaling utilizing the Hallmark gene sets^2^. Gene oncology (GO) annotations are the collaborative effort of developing and using ontologies to support biologically meaningful annotations of genes and their products, which include the biological process (BP), cellular component (CC), and molecular function (MF). Commonly, GO can be used to describe the annotation of the enriched genes in related signaling pathways and confirm the biological characteristics at the transcriptomic level. DEGs were classified using the clusterProfiler package. GO and KEGG were enriched based on the hypergeometric distribution of the GO concepts and KEGG pathways. To avoid high FDRs in multiple tests, the *q*-values of FDR control were also calculated.

### Protein-Protein Interaction Network and Module Analysis

The Search Tool for the Retrieval of Interacting Genes (STRING)^3^ (Bader and Hogue, 2003) was used for creating the protein-protein interaction (PPI) network of the DEGs and further attribute these genes to their specific biological functions, e.g., cellular component, biological process, and molecular function annotations (Dennis et al., 2003; Szklarczyk et al., 2015). Then the Cytoscape software (v3.0)^4^ was used to visualize the PPI network and identify the core DEGs in the biological regulating process. Then the KEGG pathway was analyzed for the enrichment of DEGs in the top-ranked three modules.

### TIMER Database Analysis

Immune cell infiltration analysis was performed using TIMER 2.0^5^ (Li et al., 2020). The association between the *PTEN* status of different cancers and the abundance of immune cell infiltrations were analyzed using the TIMER database to conclude the abundance of tumor-infiltrating immune cells, including B cells, CD8^+^ T cells, macrophages, dendritic cells, CD4^+^ T cells, and neutrophils.

### Statistical Analysis

All statistical analyses were conducted using Graphpad and R 3.3.0. Student’s *t*-test was used to analyze *PTEN* mRNA expression levels in cancer tissues with different *PTEN* statuses. The Benjamini-Hochberg procedure was used to adjust FDR in limma and GSEA (Mootha et al., 2003; Subramanian et al., 2005). A *p*-value < 0.05 was considered as significant. The survival curve was obtained using the cBioPortal website^6^.

## Results

### Data Information

**FIGURE 1:**
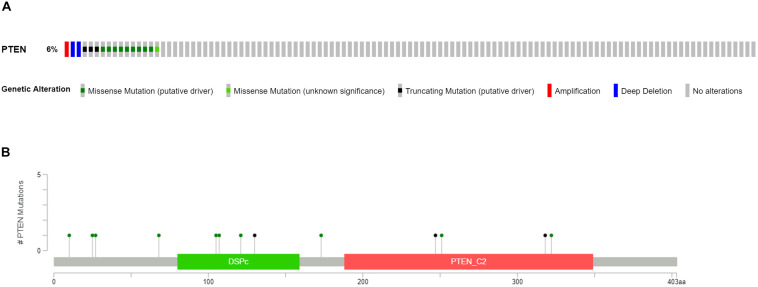
Mutation frequency **(A)** and types **(B)** of *PTEN* mutations in patients with LGG obtained from The Cancer Genome Atlas (TCGA) database.

Clinical patient information of LGGs, including the cancer tissue RNA-seq database and complete follow-up profiles, were obtained from the TCGA database. The LGG cases were divided into two groups, LGG with *PTEN* mutation (18 patients) and LGG without *PTEN* mutation (as shown in Figure 1A). Among these patients, 6% of LGG patients had mutated genes, which included missense mutations, nonsense mutations, amplifications, and deep deletions. For the *PTEN* mutation (Figure 1B), there were 13 amino acid sites of the PTEN protein that were identified as the commonly mutated sites, located at the DSPc and PTEN_C2 domains.

### Clinical Impact of Low-Grade Glioma Progression and Prognosis

Clinical information for the LGG patients can provide a profile of related characteristics. Before further bioinformatics analysis, we studied the clinical information of the included patients, as shown in Table 1. The average age (54.44, 35–74 years old) of patients with the *PTEN* mutation was higher than the wild-type patient (42.52, 14–87 years old), indicating that age may promote the mutation of the *PTEN* gene. Moreover, the histological grade (G4:G3 = 16:2) of LGG patients harboring the *PTEN* mutation was higher than the wild-type group (G4:G3 = 247:250), indicating that LGG with a *PTEN* mutation is more serious.

**TABLE 1:** Clinical characteristics of patients with low-grade glioma and their *PTEN* status obtained from the Cancer Genome Atlas database.

Characteristics	PTEN status	
	Wild-type	Mutated
**Age, years**	42.52	54.44
Range	14–87	35–74
**Gender**		
Female	224	6
Male	273	12
**Histologic grade**		
G3	247	2
G4	250	16

**FIGURE 2:**
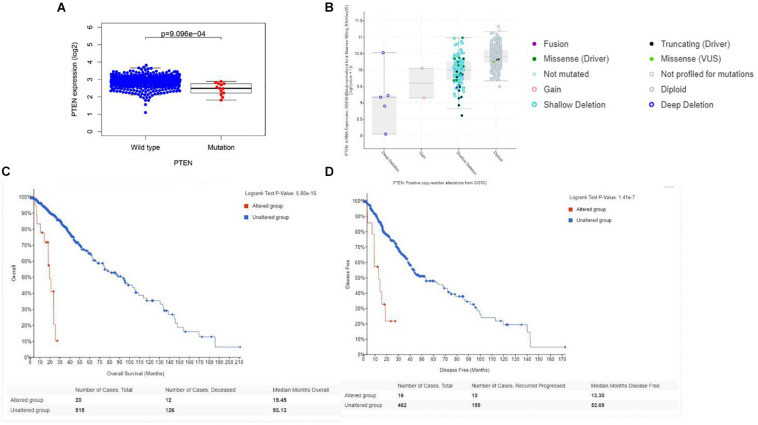
**(A)** Correlation between the *PTEN* mutation and mRNA expression; **(B)** transcriptional expression of *PTEN* dependence on the *PTEN* status. **(C)** Overall survival of LGG patient dependence on the PTEN status (alteration and wild-type). **(D)** Disease-free survival of LGG patient dependence on the PTEN status (alteration and wild-type).

Initially, the *PTEN* mRNA expression level of the wild-type *PTEN* and *PTEN* mutation groups were identified. As shown in Figure 2A, the *PTEN* expression level of the PTEN wild-type group was significantly higher than the *PTEN* mutated group. Meanwhile, the *PTEN* expression dependence on the PTEN status (as shown in Figure 2B) showed that the expression level of shallow deletion and diploid was significantly higher than the gain and deep deletion status.

**FIGURE 3:**
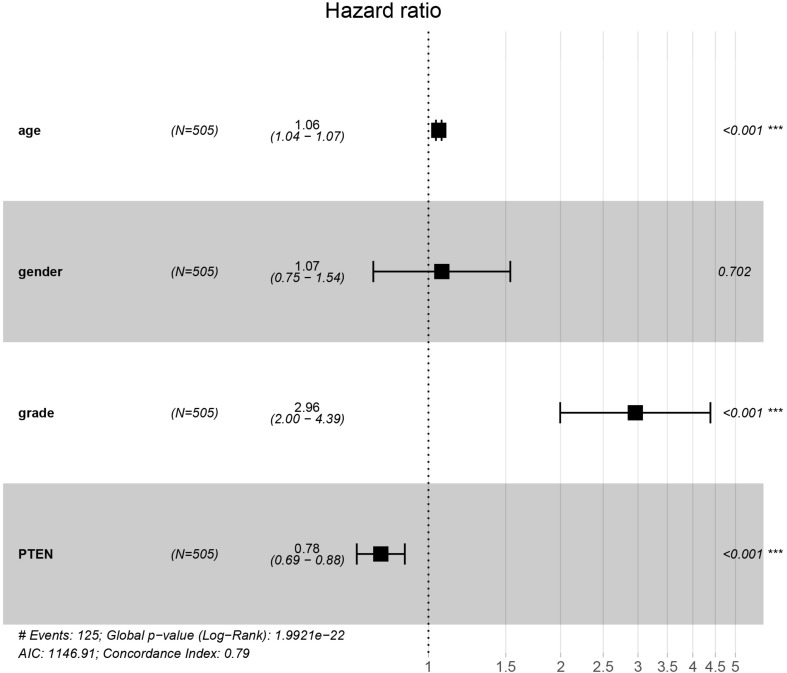
*PTEN* expression levels affected the prognosis of patients with low-grade glioma independently of tumor stage and patient age and gender.

*The PTEN* gene is known as the tumor suppressor gene, while *PTEN* mutation can decrease the inhibition of tumorigenesis. In a previous investigation, a patient with LGG recurrence suffered a poor prognosis (Liu et al., 2020). Thus, early treatment may be helpful for precise therapy in LGG patients harboring the *PTEN* mutation. To perform the Cox regression analysis of multiple factors and *PTEN* mRNA expression including tumor grade and patient age, the static results revealed that *PTEN* mRNA expression levels may affect the prognosis of LGG patients, which is independent of tumor grade, patient age, and gender (Figure 3).

### *PTEN* Status Is Correlated With Immune Cell Infiltration Levels in Low-Grade Glioma

**FIGURE 4:**
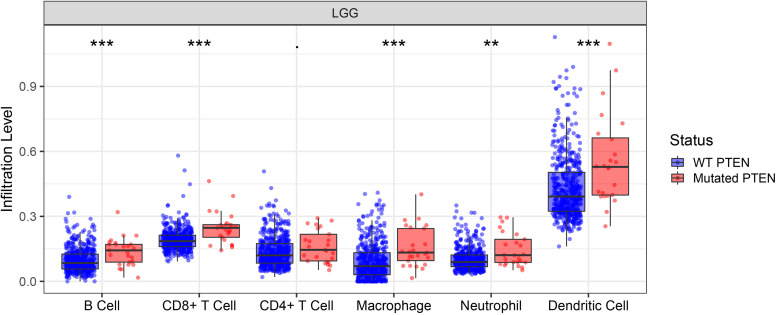
*PTEN* mutation significantly correlates with immune cell infiltration. **p ≤ 0.01, ***p ≤ 0.001.

The correlation between *PTEN* status and immune cell infiltration (including B cells, CD8^+^ T cells, CD4^+^ T cells, macrophages, neutrophils, and dendritic cells) in LGG patients were evaluated using the TIMER database. The results showed that the *PTEN* mutation is significantly and positively correlated with the infiltration of B cells, macrophages, neutrophils, CD8^+^ T cells, and dendritic cells in LGG patients (Figure 4), but not CD4^+^ T cells. Among these differential groups, the immune cell infiltration of *PTEN* mutation was significantly higher than the wild-type group.

### Gene Set Enrichment Analysis

**FIGURE 5:**
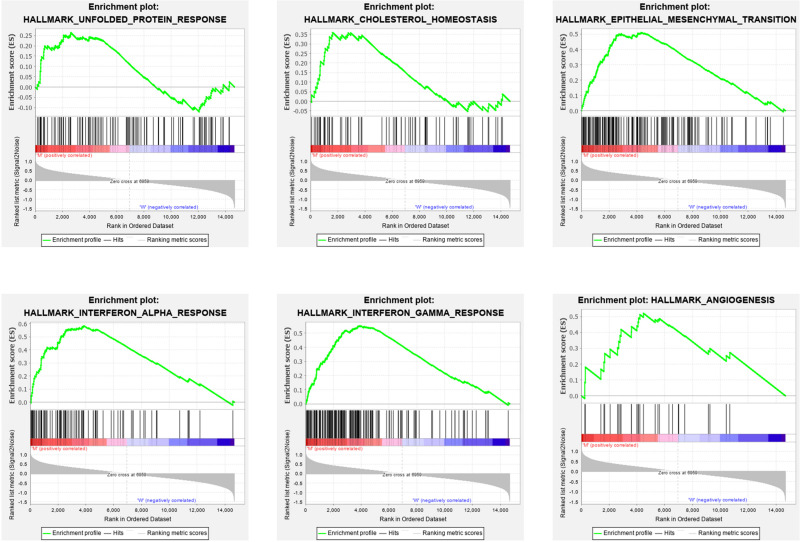
Gene set enrichment analysis results for high *PTEN* expression levels in patients with low-grade glioma.

To explore the effect of the DEGs on molecular function signaling, the GSEA analysis was employed. By performing the GSEA analysis, we identified eight significant biological function annotations, e.g., unfolded protein response, cholesterol homeostasis, epithelial mesenchymal transition, interferon alpha response, interferon gamma response, and angiogenesis (Figure 5). These annotations are the critical components in cancer cell proliferation. The enrichment results indicated that the *PTEN* mutation may play a pivotal role in various pathways involved in cancer cell migration, metabolism, and immune response regulation.

### Identification of Differentially Expressed Genes

**FIGURE 6:**
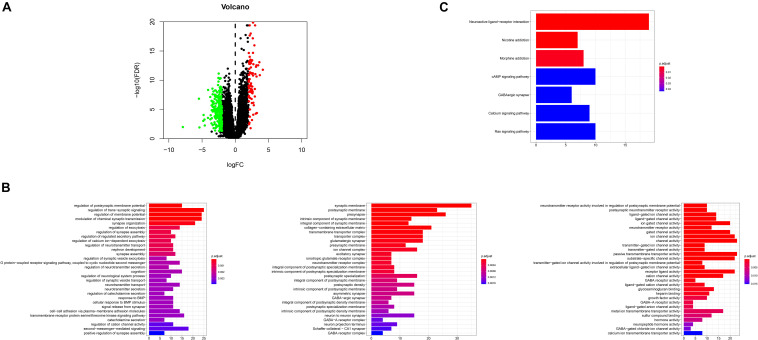
**(A)** Volcano plot for the differentially expressed genes (DEGs); **(B)** GO enrichment terms of the DEGs, **(C)** KEGG pathway analysis of the DEGs.

DEGs were identified by querying the RNA-seq datasets from the *PTEN* mutation (*n* = 18) or wild-type *PTEN* groups (*n* = 498). Here, 352 genes were identified as DEGs based on the criteria of | log2FoldChange | ≥ 1.0 and *P* < 0.05 (as shown in Figure 6A). Among these DEGs, 91 genes were upregulated and 261 genes were downregulated. Meanwhile, we also explored the correlation between PTEN expression and tumor-related biomarker expression (including Nf1, H3F3A, CDKN2A, IDH1, and FGFR1/2) as shown in Table 2. The NF1 expression level was highly positively correlated with PTEN expression (Spearman’s efficiency *R* = 0.405).

**TABLE 2:** Correlation between PTEN expression and tumor-related biomarkers.

Gene	Spearmen’s correlation	*p*-value	*q*-value
NF1	0.405	9.26*e*−22	2.72*e*−20
H3F3A	–0.172	8.753*e*−5	2.215*e*−4
CDKN2A	–0.131	2.944*e*−3	5.793*e*−3
IDH1	–0.102	0.0212	0.0354
FGFR1	–0.0845	0.0555	0.0845
FGFR2	–0.151	5.952*e*−4	1.319*e*−3

### GO and KEGG Analyses of Differentially Expressed Genes

In order to explore the biological effect of the dependence of these 352 DEGs on *PTEN* status, we performed GO and KEGG pathway analyses. The GO analysis of the DEGs (Figure 6B) suggested that they were enriched during the regulation of the postsynaptic membrane potential, transsynaptic signaling, the regulation of membrane potential, modulation of the chemical synaptic transmission, synapse organization, synaptic membrane, postsynaptic membrane, pre-synapse, integral components of the synaptic membrane, regulation of the neurotransmitter receptor activity involved in the regulation of the postsynaptic membrane potential, postsynaptic neurotransmitter receptor activity, and ligand-gated ion channel activity. Moreover, the DEGs of the KEGG analysis were enriched in nicotine addiction, morphine addiction, the cyclic adenosine monophosphate (cAMP) signaling pathway, and neuroactive ligand-receptor interaction (Figure 6C).

### Module Screening

**FIGURE 7:**
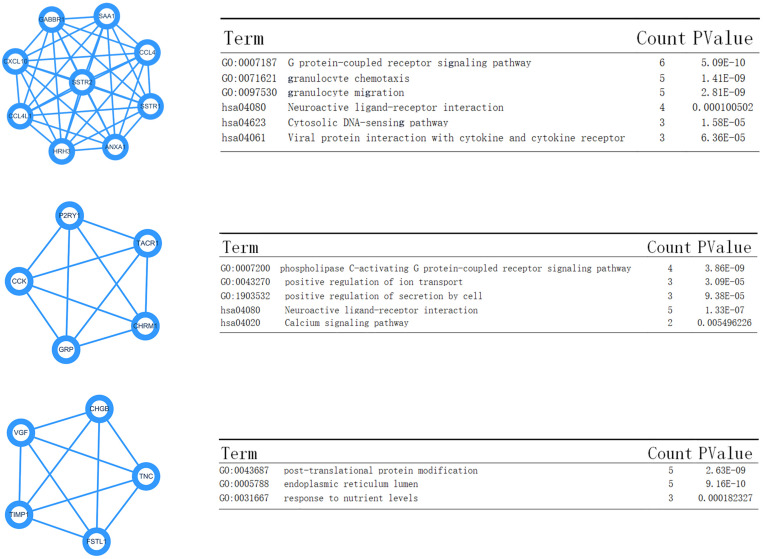
Top three modules from the Pixels Per Inch (PPI) network—**(A,B)** PPI network and GO and KEGG analyses of module 1; **(C,D)** PPI network and GO and KEGG analyses of module 2; **(E,F)** PPI network and GO and KEGG analyses of module 3.

Data created by the STRING database were filtered, and the mutual effect and central genes within the DEGs were studied. The top 10 genes were confirmed to be central genes. These were confirmed as hub genes and included *PSSTR2*, *GABBR1*, *SSTR1*, *CXCL10*, *CCL4*, *ANXA1*, *SAA1*, *CCL4L1*, and *HRH3*. *SSTR2* exhibited the highest degree of nodes among those genes with nine. In the PPI network, the modules of genes were confirmed using the MCODE plug-in in Cytoscape. The top three modules of the GO and KEGG pathways were chosen for analysis (Figure 7). The enrichment results suggested that the genes in modules 1–3 were predominantly associated with the G protein-coupled receptor signaling pathway, coupled to the cyclic nucleotide second messenger, endothelial cell activation, gamma-aminobutyric acid (GABA) receptor activity, cAMP signaling pathway, phospholipase C-activating G protein-coupled receptor signaling pathway, neuroactive ligand-receptor interaction, and post-translational protein modification.

## Discussion

The PTEN protein acts as a tumor suppressor, which inhibits the down-stream proteins when performing its suppressing function (Maehama et al., 2001; McGirt et al., 2005). The bioactivity of the PTEN protein is highly dependent on the subsequent antagonism of the PI3K/AKT pathway. However, some literature has reported that PTEN can function through AKT-independence (Freeman et al., 2003). Consequently, it is necessary to explore the biological functions associated with the *PTEN* status. In this study, we carefully evaluated the critical role of *PTEN* mutation in LGG progression and prognosis, which may provide therapeutic scope for precise LGG therapy.

Firstly, the clinical analysis (Figure 1A) results showed that 6% of patients with LGG harbored *PTEN* mutations, including four types of mutations (missense mutations, nonsense mutations, amplifications, and deep deletions). Furthermore, the survival curve clearly revealed that patients with the *PTEN* mutation suffered a poorer prognosis, a lower survival rate, and greater disease recurrence than the wild-type group (Figures 2C,D). On the basis of the clinical results, early clinical intervention for LGG *PTEN* mutation groups would be helpful for improving the patient survival period.

Second, the Cox analysis revealed that the mRNA expression level of the *PTEN* mutation group was lower than the wild-type group (*P* < 0.01). And shallow deletion and normal diploid types of *PTEN* mRNA level were also higher than the deep deletion and gain status (Figure 2B). Considering the *PTEN* mutation types (missense), a mutation of *PTEN* led to the dysfunction of tumorigenesis suppression. Meanwhile, we also found that the PTEN expression level was related with some tumor-related biomarkers (Table 2).

Immune cell infiltration can affect the tumor occurrence, progression, and prognosis, owing to the effect of the tumor microenvironment (Chaffer and Weinberg, 2011; Deng et al., 2020). The tumor microenvironment is highly associated with immune cell infiltration. Consequently, exploring the immune cell infiltration level may provide more scope on the tumor progression and immune status. Here, we identified the role of immune cell infiltration in *PTEN* status using the TIMER database (Figure 4). By further analysis, B cells, neutrophils, CD4+ T cells, macrophages, CD8+ T cells, and dendritic cells were more significantly abundant in the *PTEN* mutation group than the wild-type *PTEN* group. Higher immune cell infiltration meant that the complex immune microenvironment may induce a serious progression status. Therefore, these results revealed that some specific genes or signaling may lead to immune cell infiltration in the *PTEN* mutation group.

Finally, we explored the role of critical molecular annotations that led to the poor prognosis of *PTEN* mutation LGG patients (Figures 5, 6). The top six annotations of GSEA were associated with various cancer-related pathways, e.g., epithelial mesenchymal transition, interferon gamma response, interferon alpha response, cholesterol homeostasis, unfolded protein response, and angiogenesis. These molecular functions promoted tumorigenesis (epithelial mesenchymal transition) and enhanced drug resistance (unfolded protein response). By deeply affecting these signal pathways, LGGs with *PTEN* mutations can lead to a higher tumor grade and poor survival (Figures 2C,D).

After the identification of DEGs (Figure 6A), the GSEA analysis on the biological function levels were carefully studied (Figures 6B,C). The GO annotations showed that the top five ranking annotations were mostly associated with the signal transduction process, for example, the regulation of postsynaptic membrane potential, synaptic membrane, and ligand-gated ion channel activity. These annotations were “neuron”-related signaling and these molecular functions may affect cell metastasis. Among them, epithelial mesenchymal transition, a complex biological process, contributed to metastasis, wherein the genetic and epigenetic events caused the epithelial cells to acquire a mesenchymal gene activity signature and phenotype (Subramanian et al., 2005; Li et al., 2020). The GO analysis results showed that 352 DEGs could be attributed to the top three GO annotations: growth factor activity, GABA receptor activity, and neurotransmitter secretion. The enriched annotations of growth factor activity and neurotransmitter secretion were consistent with immune cell infiltration.

Moreover, the KEGG enrichment results confirmed the GO analysis, because the top ranking pathways were neuron-related molecular functions and tumor-related pathways (Ras signaling pathway). The PPI network analysis also provided four key nodes by STRING analysis (Figure 7). These node networks were highly associated with the protein modification process.

## Conclusion

In summary, we systematically investigated the *PTEN* mutation condition with LGG poor prognosis and immune cell infiltration. These results revealed that a PTEN mutation can promote the tumorigenesis process and lead to more immune cell infiltration. Thus, our results showed the importance of *PTEN* status in disease progression and revealed that it may become a useful biomarker for diagnosing LGGs.

## Data Availability Statement

Publicly available datasets were analyzed in this study. This data can be found here: TCGA.

## Author Contributions

All authors contributed to the literature investigation, data collection, writing the manuscript, providing useful discussion of its content, and undertaking reviews or revising the manuscript before submission.

## Conflict of Interest

The authors declare that the research was conducted in the absence of any commercial or financial relationships that could be construed as a potential conflict of interest.

## Footnotes

1 https://portal.gdc.cancer.gov

2 https://www.gsea-msigdb.org/gsea/msigdb/index.jsp

3 https://string-db.org/

4 https://cytoscape.org/

5 http://timer.cistrome.org/

6 https://www.cbioportal.org

## Abbreviations

DEG: Differentially expressed genes
FDR: False discovery rate
GABA: Gamma-aminobutyric acid
GSEA: Gene set enrichment analysis
LGG: Low-grade gliomas
mRNA: messenger ribonucleic acid
PPI: Protein-protein interaction
STRING: Search Tool for the Retrieval of Interacting Genes
TCGA: The Cancer Genome Atlas.
